# On the edges of medicine – a qualitative study on the function of complementary, alternative, and non-specific therapies in handling therapeutically indeterminate situations

**DOI:** 10.1186/s12875-019-0945-4

**Published:** 2019-04-23

**Authors:** Agnes Ostermaier, Niklas Barth, Antonius Schneider, Klaus Linde

**Affiliations:** 1 Technical University of Munich, TUM School of Medicine, Institute of General Practice, Orleansstrasse 47, 81667 Munich, Germany; 2 Ludwig-Maximilans-Universität Munich, Institute of Sociology, Konradstr. 6, 80801 Munich, Germany

**Keywords:** Primary health care, General practice, Complementary and alternative medicine, Placebo, Medically unexplained symptoms, Minor ailments

## Abstract

**Background:**

In routine practice, general practitioners (GPs) see many patients for whom treatment might not be necessary, or evidence-based treatments are not available, yet often a treatment is prescribed. We denote such situations as therapeutically indeterminate. We aimed to investigate 1) whether therapeutically indeterminate situations play a role in the accounts of GPs in their practical work; 2) the role of complementary and alternative medicine (CAM) modalities or non-specific therapies, and of other strategies used in handling therapeutically indeterminate situations; and 3) factors associated with preferences for specific strategies.

**Methods:**

We performed semi-structured, individual face-to-face interviews with 20 purposively sampled, experienced GPs from Bavaria, Germany. A grounded theory approach was used for data analysis.

**Results:**

Participants reported that therapeutically indeterminate situations recur often in their daily practice. Professionally legitimate strategies such as empathetic consultations without providing a treatment intervention did not seem to suffice for coping with all of these situations. CAM treatments were used frequently, but motives varied. While some participants were convinced that these treatments were active and effective, others were uncertain or had doubts and used them as a relational tool, as a non-specific treatment or as a beneficial placebo. Conventional drugs were also used in a non-specific manner or despite doubts regarding the risk-benefit ratio. The extent to which GPs felt responsible for offering solutions in therapeutically indeterminate situations seemed to influence their preference for specific strategies.

**Conclusion:**

Our results demonstrate the important role of CAM and the somewhat smaller role of non-specific therapies for German general practitioners in dealing with therapeutically indeterminate situations. The concept of therapeutically indeterminate situations may be helpful in better understanding why many general practitioners treat patients in situations where treatment does not appear to be clearly indicated.

**Electronic supplementary material:**

The online version of this article (10.1186/s12875-019-0945-4) contains supplementary material, which is available to authorized users.

## Background

In the WONCA (World Organization of National Colleges, Academies and Academic Associations of General Practitioners/Family Physicians) Europe definition of 2011, general practice is described explicitly as an “academic and scientific discipline” [1]. However, in routine practice many general practitioners (GPs) also use treatment approaches which do not seem to conform with this ideal. For example, in some countries a considerable proportion of physicians prescribe complementary and alternative medicine (CAM) treatments [2, 3]. From a biomedical perspective, many CAM modalities lack biological plausibility and evidence of effectiveness. The use of CAM seems to be particularly prevalent among German physicians. In a national survey [4], 85% of GPs reported using at least one CAM treatment modality once weekly or more often, with herbal remedies (77%), vitamins and supplements (41%) and homeopathic remedies (32%) being most frequent. Another example of scientifically and professionally problematic procedures is the widespread use of “non-specific interventions” (also sometimes called “impure placebos”) among GPs (see [5] for a systematic review). These are potentially active interventions, which the GPs themselves do not consider to have a specific effect on the condition being treated. Typical examples in surveys are antibiotics in patients with common colds or vitamins in patients without deficiency [5–7]. In a survey form the United Kingdom, 75% of GPs reported that they prescribe non-specific treatments at least once a week [6]; in a German survey this was reported by 30% of GPs [7]. In rare circumstances, a minority of GPs even use “pure” placebos, such as saline injections or placebo pills [5–7].

Why do physicians, in general, and GPs, in particular, use these strategies? We assume that they are attempts to manage *therapeutically indeterminate situations.* This concept was only vaguely defined when we started the study described below but was honed by the accounts of participants. Therapeutically indeterminate situations seem to be characterized by two sets of conditions: firstly, there is a desire for medical treatment, either by the patient or the physician, or both. Secondly, either such a treatment is not (unambiguously) necessary from a medical perspective, a professionally accepted treatment is not available, or an existing effective treatment is not acceptable to the patient. Such situations occur frequently in encounters with patients suffering from minor illnesses, non-specific complaints, medically unexplained symptoms, or long-lasting complaints associated with chronic diseases. Our assumption was based on three strands of evidence: 1) the limited number of qualitative studies addressing the issue of “non-scientific prescribing” in a broader manner [8–12], 2) the quantitative work on the widespread use of CAM and non-specific treatments partly cited above, and 3) sociological work on indeterminateness [13–15]. Among the studies on “non-scientific prescribing” Comaroff’s seminal study among 51 Welsh GPs from 1976 [8] had a great influence on our thinking, and caused us to wonder whether her findings still apply today. Comaroff described how physicians had internalised a professional ideal which requires that any treatment should be specific in effect and administered or prescribed only when necessary. However, this ideal conflicted with the realities of routine practice. Since they only saw unselected patients, GPs faced considerable uncertainty but still needed to make decisions. Making a firm diagnosis in general practice was often impossible or unnecessary, implying that the basis for choosing a specific treatment was weak. On the other hand, participants usually believed that patients expected a clear diagnosis and treatment [8]. From an academic point of view, the optimal solution in such situations on the edges of medicine is an empathetic consultation without prescribing a treatment [16]. However, many practitioners seem to resort to other solutions.

Comaroffs’ interpretation of her study findings builds on the analysis of the medical system by Parsons [13]. According to Parsons there is an “optimistic bias” in medicine which prefers treating (or acting in general) over not treating (or not acting). In situations of uncertainty and emotional concern, physicians tend to convey their concern for the patient by doing something practical, even if scientific medicine does not provide a solution. In the German sociological discourse, indeterminateness (German “Unbestimmtheit”) is an important topic [14]. This concept emphasizes that decisions are necessary only when optimal solutions are not self-evident. Compared to uncertainty, indeterminateness is not only a problem but also a basis for productive and creative strategies. Given the level of uncertainty in day-to-day practice, GPs should be almost specialists in dealing with therapeutic indeterminateness [15].

We aimed to investigate 1) whether therapeutically indeterminate situations play a role in the accounts of GPs on their practical work; 2) what role CAM, non-specific therapies and placebos have in therapeutically indeterminate situations, and which other strategies are used; 3) which factors are associated with preferences for specific strategies.

## Methods

We performed a qualitative study with semi-structured interviews (performed between July 2015 and December 2016) of GPs in Bavaria, Germany. The study protocol was approved by the ethics committee of the Medical Faculty of the Technical University of Munich (reference number 450/15 s).

### Recruitment and sample

Twenty experienced GPs (see Table 1) providing first-line primary care within the German social health insurance system participated in interviews. Six participants were recruited from practices cooperating with the Institute of General Practice of the Technical University of Munich. The remaining participants were mostly recruited with the help of two highly experienced GPs with good connections to regional colleagues. We aimed to recruit GPs managing indeterminate situations in different manners. The most feasible way to approximate this goal seemed to us to recruit GPs representing a broad spectrum of attitudes towards CAM therapies ranging from “skeptics” (GPs aiming to exclude CAM treatments as far as possible) over “pragmatists” (GPs applying CAM treatment more or less but without considering them a core component in their practice), and “convinced CAM users”. Limited pre-interview information on CAM use was obtained from practice websites and personal contacts. Furthermore, we took account of gender and practice location. All participants gave written and oral informed consent.

**Table 1:** Characteristics of participating general practitioners (*n* = 20)

Female gender	8
Age group	
41 to 50 years	10
51 to 60 years	5
61 to 70 years	5
Practice location	
City or suburb	4
Town	10
Village	6
Specialized as GP since more than 10 years	14
Practice owner or joint partner	18
At least one CAM qualification	9
Acupuncture	6
Naturopathy	5
Homeopathy	5
Chirotherapy	2
Additional qualification emergency medicine	8

### Data collection

All interviews were conducted face-to-face with a single GP (19 by AO, 1 by NB and KL). The median duration was 52 min (range 34 to 72 min). Interviews were informed by a topic guide (see Additional file 1), but we aimed to encourage the interviewees to develop the topics themselves as far as possible. Topics to be addressed included typical features of their practice and how they had changed over the years; attitudes to and use of CAM treatments, non-specific treatments and placebos, and if such treatments were not used, how practice was managed without; scientific orientation and legitimisation conflicts; and their views on their role as a physician and medical doctor. Indeterminateness was not addressed explicitly by the topic guide. Interviews were audio-taped, transcribed verbatim, and pseudonymised.

### Analysis

Our analysis was inspired by grounded theory [17, 18]. Our primary perspective was functionalistic with the key question being: what problems are solved by the use of CAM, non-specific treatments and placebos. The evaluation framework of the interview data followed methodological principles of system-theoretical hermeneutics. Through the functionalist heuristic, narrative patterns were read as communicative solutions to self-imposed problematisations [19].

All interviews were read by AO (at the time of the study a medical student with previous experience as a CAM practitioner) and KL (a clinical epidemiologist with 25 years’ experience in quantitative research on CAM and placebo). A selection of interviews were also read by NB (a sociologist specialised in systems theory) and AS (a GP and professor of general practice). Analysis started early in the data collection phase. In a first step, open coding was performed by AO. Documentation was supported by using MAXQDA 12 software. Units of coding could be single sentences but more often were several sentences to maintain (and code) structural references. The codes were continuously sorted and compared until categories with similar information emerged. Categories were revised if necessary based on new data. In a second phase of axial coding, categories were consolidated and we tried to identify how categories related to each other, also with respect to the assumptions described above. Selective coding was not formally separated from axial coding, but in the third phase we aimed to construct a story line around the perception of indeterminateness and the functional strategies for dealing with it. Throughout the analysis, we followed a constant comparative approach. Transparency and trustworthiness in open coding was observed by the senior author who independently reviewed the coding framework, and then through discussion with the sociologist. Written memos, reflective notes and mind maps supported the analysis. Findings were reported twice to groups of GPs to obtain feedback on the practical relevance of the categories developed.

## Results

### The dominance of pragmatism

A first finding which impressed and surprised us in the interviews with our participants was the extent of pragmatism when dealing with difficult or unclear situations. In our context, pragmatism meant that GPs aimed to balance biomedicine, the wishes and the psychosocial situation of patients, and the reality of a busy primary care practice in a quite permissive manner. One consequence of the dominance of pragmatism was that our aim of having similar numbers of CAM “skeptics”, “pragmatists” and “convinced CAM users” was not achieved. During interviews it became clear that the attitudes of practitioners did not always match with the limited information available or self-classification provided at recruitment. Most “pragmatists” used and to various degrees believed in the efficacy of some CAM therapies but rejected others. Even the more skeptical physicians used at least some herbal medicines. In our attempt to broadly categorise interviewees based on the general tendencies in their statements, we considered only one GP a true skeptic and four GPs as clearly convinced CAM users. The rest were pragmatists on a wide and vague spectrum from rather skeptical to very open to CAM.

### Biomedical clarity versus uncertainty and indeterminateness

When talking on their work in general practice participants, including convinced CAM users, spontaneously emphasized that the basis of their daily work is conventional medicine (not CAM). At the same time, they experienced general practice as very special. Together with the continuity of the patient-physician relationship and diagnostic uncertainty the frequency and relevance of what we call therapeutically indeterminate situations was a central theme in this respect.

GPs reported examples from a wide spectrum of disorders reaching from “non-specific complaints” and “minor illnesses” such as “sore throat” over sub-acute and chronic problems such as “irritable bowel syndrome” to accompanying “patients with cancer seeking something in addition to conventional treatment”. When starting general practice, GPs often felt insufficiently prepared to deal with the numbers of patients suffering from ill-defined problems or from problems for which they did not have convincing solutions. However, they had internalised the conviction that they should have an answer to each medical problem.

*… The unfamiliar thing was also that many people come with complaints that you cannot classify initially, that you haven’t heard about at medical school. And well, non-specific disorders for which you need to have an answer. Or you think you have to have an answer – my initial reaction was, you always have to have an answer. (GP 20)*

*The easiest case is when I recognise a clear problem, an acute gout attack, for example, and I know exactly how to treat it according to guidelines, what to check and so on. More frequently, however, it happens that you fail to address the patients’ complaints and that there is nothing which really fits. ... If someone presents with a sore throat, I try to find out whether there is a scientifically exact cause, a streptococcal angina, an Epstein-Barr virus infection, and so on. And if there is such a cause, I try to treat according to guidelines or textbooks. But if I don’t find such a cause, then other things [with limited evidence] start to become of interest. (1)*

The clear-cut cases allowed GPs to identify a defined pathology and to proceed according to scientific reasoning and guidelines. But in the perception of our participants there were many cases which were less clear or unclear. The clarity of biomedical knowledge learned at medical school and in the hospital was contrasted with problems for which this knowledge did not fit. Over the years, participants had developed functional strategies to deal with these cases.

### Functional strategies used to deal with therapeutically indeterminate situations

Participants reported a variety of approaches for dealing with therapeutically indeterminate situations. A general basic approach was *the therapeutic encounter instead of a medical treatment.* In line with the dominance of pragmatism, the remaining strategies cover a spectrum from convinced use of CAM, the use of CAM and conventional drugs as non-specific treatments, to symptomatic treatment. The general repertoire of GPs typically consisted of two or more of these strategies with variable preferences and boundaries between the strategies being porous. We also asked GPs explicitly whether they made use of pure placebos, such as saline injections or prepared pills without an active ingredient (which can be obtained in German pharmacies). However, pure placebos were used only rarely by a minority of participants. The main reason for rejecting or avoiding placebo use was the discomfort about actively deceiving patients.

#### The therapeutic encounter instead of medical treatment

In their accounts, almost all participants spontaneously reported that a relational approach where they provided no medical treatment but listened and consulted empathically was a crucial and desirable strategy for dealing with therapeutically indeterminate situations.

*Often it’s sufficient when you reassure the patient that it’s nothing dangerous, that it will pass and that there probably is no need for treatment. (4)*

GPs reported that the aptitude for watchful waiting increased with increasing practical experience. Respecting the patient’s suffering, ruling out major somatic disease, taking psychosocial aspects into account, and providing re-assurance were key elements when GPs tried to avoid unnecessary diagnostic and therapeutic interventions. In using this strategy for dealing with indeterminateness, physicians openly acknowledge the lack of an indication for treatment or the lack of a treatment option, and do not comply with the desire for treatment. However, none of the participants were able to manage their practice exclusively with this approach.

#### CAM as an active and effective therapy

Convinced CAM users were generally certain that their treatments were specifically active, but also a number of pragmatic GPs considered selected CAM treatments (e.g. specific herbal medicines) as being active. In this way, CAM expanded the spectrum of “true” therapeutic options, complied with the expectations of patients, and thus reduced or even removed indeterminateness.

*... the medicine I learned at medical school offers no options for many patients, and I don’t know how to help them. ... Homeopathy allows me so many more treatment options that I otherwise wouldn’t have. (7)*

#### CAM as a specific relational tool

Several general practitioners reported that CAM approaches improve communication and outcomes by enabling a better connection with patients. Homeopathy was used repeatedly as an example in this respect:

*There I see an advantage in the approach [homeopathy]. It’s very good for listening, very good for information gathering, something I would actually often wish for conventional medicine. ... And I ‘ve also experienced this personally, if I establish a connection with the patient, in conventional medicine as well as in homeopathy as well as in all other things, if it clicks, and there is a connection, then medicine works. No matter what I prescribe. (3)*

In these cases CAM was valued for strengthening the therapeutic alliance. It allowed GPs to find common grounds with patients and to provide explanations that accorded with their particular lifeworlds. Irrespective of whether a CAM treatment had an intrinsic biological effect it provided participants with a specific relational tool filling a gap left by conventional medicine and improving outcomes. Furthermore, some participants mentioned that openess to CAM increased trustworthiness when prescribing conventional treatment to patients sceptical about conventional drugs.

#### CAM for a first therapeutic intervention avoiding harm

Participants frequently reported that they prescribed or recommended CAM treatments or household remedies to avoid potentially harmful conventional treatments. Usually, GPs reported positive previous experiences with the specific treatment, but this did not always mean they were convinced that it had relevant intrinsic effects. The demarcation as to whether CAM was used as a specifically active or as a non-specific treatment became vague.

*For example, there are many people with insomnia. I find it very difficult to treat people without prescribing them something to which they could become addicted. For example, sometimes I give valerian, and that helps some people and it is enough for them. They just have to know there is something they can take, then they can sleep. (5)*

GPs who were uncertain about the biological activity preferred treatments that, in their view, had some plausibility over other CAM therapies they considered less plausible or entirely implausible. GPs sometimes presented the treatment as a first attempt and communicated their uncertainty about effectiveness to some degree to the patients. If CAM treatment failed, more powerful conventional treatments would be available.

#### CAM as a non-specific treatment for eliciting placebo effects

In a few cases GPs used a CAM treatment although they explicitly thought it had only placebo effects. An extreme example is a GP prescribing a homeopathic remedy for vertigo:

*Interviewer: Does the subject of placebo play a role in your practice?*

*GP: Of course, quite considerably, except that I don’t use the word “placebo”, of course.*

*Interviewer: How do you do it then?*

*GP: “I have something homeopathic”. … I have my remedies, the most usual and frequent one is X [a homeopathic combination for vertigo]. … Vertigo is really common. Obviously, you first have to check what kind of vertigo it is. But there are many types of vertigo or dizziness which you cannot treat with drugs or conventional medicine. … But X is really great, with the ritual surrounding it, obviously the instructions for taking it, and homeopathy is something good, it’s well known, it has a good reputation among patients. … And it helps many! Even if it’s not gone, it makes it easier to endure. (6)*

In such cases GPs exploited the positive attitudes of patients towards CAM. The GP’s belief that the treatment is considered a placebo was not only hidden, but the GP actively promoted the homeopathic remedy. In fact, he was convinced that it would be associated with beneficial placebo effects. Given its specific contextual characteristics the homeopathic remedy could not have easily been replaced easily another (placebo) intervention.

#### Other reasons for prescribing non-specific treatments

A variety of other reasons were occasionally mentioned for using CAM or conventional drugs as non-specific treatment. For example, in a patient with vertigo a drug was prescribed to buy time until psychotherapy started to help. If expectations of patients to receive a treatment were perceived as strong, GPs gave in when risks were considered low and time scarce. GPs did not always inform patients that they did consider these treatments unnecessary or ineffective.

#### Prescribing antibiotics to avoid conflict and save time

Regarding non-specific treatments, the frequent accounts on non-indicated use of antibiotics were a special case. Here GPs usually reported explicitly that they had tried to communicate that antibiotic treatment was not needed but they sometimes complied with the wish of patients to avoid conflict or save time. This was experienced as a (mostly minor) internal conflict.

*No matter how hard you try ... if the patient comes with the idea that they need an antibiotic, then you can talk as much as you want, but you can’t persuade them otherwise. (9)*

As reported above, participants often preferred to avoid antibiotics by prescribing herbal or other complementary treatments if these were acceptable compromises for patients. Some GPs using CAM only in a very limited manner also mentioned that it was difficult from them to handle such situations because they were either not familiar or did not believe in these therapies.

*Maybe I also lack the know-how [in such situations] because I can’t do homeopathy. (9)*

#### (Stretching the indication for) symptomatic treatment

From our analysis we gained the impression that symptomatic treatments (e.g. nonsteroidal anti-inflammatory drugs) also seemed to be an important tool in handling therapeutically indeterminate situations. Symptomatic treatment was addressed by participants in an indirect manner in two ways.

A few GPs linked the justification of symptomatic treatments with self-critical statements that their use might not always be justified. Our most CAM-skeptic participant characterised his approach as follows:

*You have a cold now and you have a cough that is really really uncomfortable. You have to cough all night, and you’re coughing even here during the day as well. It's not dangerous, but I can understand that it’s distressing, that you’re in pain, and you can’t sleep, your wife can’t sleep. Can we alleviate the symptoms? Then I can say: you can take codeine; it’s a good cough suppressant. (2)*

The use of symptomatic treatment is justified by emphasising repeatedly what a burden the symptoms are for the patient. Other more pragmatic GPs preferred to prescribe herbal medicine in such a situation before using codeine. Later in the interview, the GP quoted above himself described his concern that he might have used symptomatic treatment too frequently:

*Well, for example, I'm somebody who always gives diclofenac way too much, I know that too, I also have a stomach ache every time I do - no, not a stomach ache, but I prescribe diclofenac more often than one should. (2)*

More frequent were statements justifying the use of CAM or non-specific treatments in order to avoid potential harm caused by symptomatic treatment (see above). This suggests that whether GPs considered a specific treatment indicated, depended also on its potential side effects. It seems that even GPs who aimed to avoid CAM or non-scientific prescribing faced situations in which they wanted to prescribe a treatment although an evidence-based option was not available or treatment was not unambiguously necessary. While pragmatic physicians preferred CAM in these situations more skeptical GPs used symptomatic treatment. Such symptomatic treatment in situations where the balance of risk and benefit is not unequivocal could be considered an approach to reducing or explaining away indeterminateness.

### Important factors associated with preferences for specific strategies

#### Perceived responsibility and the shift from disease-centered to person-centered care

In the accounts of several physicians, the experience of indeterminateness and the preferences for handling therapeutically indeterminate situations were closely linked with the question of whether a patient’s problem fell into their area of responsibility. The following quotation combines several elements reported by other participants in a single “story” and explains why our participants often wished to find an individual solution to the patient’s problem:

*That was the traditional developmental process, I see the same thing now with our registrars. When they enter general practice, they come from the hospital, from an environment where people are really seriously ill. ... In general practice, there are suddenly these non-specific disorders, and you sit in front of the patient and you have the feeling, ... treatment isn’t necessary here, there is nothing wrong with them, or whatever. Until you finally realize, well, so there is probably just as much suffering in irritable bowel syndrome as with a patient in a hospital with ulcerative colitis and 15 bloody stools a day. So the spectrum changes, in the end the suffering or the extent of suffering remains the same. And that’s how it was for me, I was just a completely technocratic physician when I first went into practice and I have, I believe, now learned that there are a lot of non-specific disorders that cause a lot of suffering. ... [The patients] want something to be done or to get better. There you have to work together somehow with the patient. And this experience is also new. When you're in the hospital, you see the patient for 14 days and then never again, and now you accompany them in the long-term. This, in turn, is something that I really like about general practice ... And this accompaniment, I feel, often means that one should not just stand there empty-handed. (13)*

The professional self-conception of participants was initially shaped by medical school and the hospital setting where they had dealt with serious illness. When coming into general practice some tended to consider patients without defined and severe pathologies as not suffering from anything real. Over the years, this benchmark changed: an objectively verifiable pathology was no longer a premise for feeling responsible as a physician – subjective suffering was sufficient. While in the hospital the patients were forgotten when leaving the ward, as GPs they now had to accompany over longer periods, often for many years. Based on this perceived responsibility, many participants seemed to feel they should meet the patients’ wish for some sort of treatment rather than send them away done empty-handed.

The question of responsibility was automatically a question of demarcation. Limits were drawn more narrowly by participants being more skeptical to CAM:

*…when the limits are reached, when medicine has nothing to offer, or where I think no action is needed, then I say so. … for me, medicine does not fail when I have to say, sorry, but we don’t have anything to offer here. (2)*

With increasing pragmatism, the boundaries became permeable. Participants, who were open to CAM, seemed to feel more responsible for problems at the edges of medicine than more sceptical GPs, who saw their task as working within the confines of a conventional medical model.

#### Patient selection

Our interviews suggest that there is an association between perceived responsibility for problems at the edges of medicine, convinced CAM use and patient selection mechanisms. The majority of participants at some point made statements of the following kind:

*…every doctor has the patients he deserves. (12)*

Patient selection seemed to be a continuous and dynamic adaptive process. On the one hand, it was influenced by the style, preferences and attitudes of the physician. On the other, the preferences and wishes of the patients shaped the style of the GPs. Practice location (e.g., in a rich suburb or in a worker’s community) and, in early years, the style of the previous owner of the practice also played a part. Several GPs mentioned that they noticed patient selection more when standing in as holiday replacements for other GPs. Sometimes, offering CAM treatments or not was actively used to influence patient selection. For example, a pragmatic participant stated:

*Initially we had thought we should also use acupuncture and other (CAM) things. But we don’t have the time. And we don’t want to. … CAM attracts a specific group of patients who we do not want in our practice, who take up a lot of time, and who have a lot of psychosomatic problems. About a third of our patients suffer from psychosomatic problems anyhow… we like these patients … but a third is enough. (12)*

GPs reported that patients attending practices offering a lot of CAM tended to be younger, more critical towards conventional medicine, were sometimes not really ill, and less often old and multi-morbid. GPs considered using therapies they personally prefer, and a patient selection resulting from their preferences as a positive process which in turn made their work easier and more satisfying.

## Discussion

### Summary of main findings

The experienced German general practitioners participating in our study considered situations that were, according to our definition, therapeutically indeterminate, common and typical of primary care. Scientifically legitimate strategies such as empathetic consultations without providing a treatment intervention did not seem to suffice for coping with all of these situations. CAM treatments were used frequently, but motives varied. While some participants were convinced that their treatments were active and effective, others were uncertain or had doubts and used them as a relational tool, as a non-specific treatment or as a beneficial placebo. Sometimes conventional drugs were also used in a non-specific manner or despite doubts regarding the risk-benefit ratio, but the majority of participants preferred CAM treatments in such situations. The extent to which GPs felt responsible for offering solutions seemed to influence the strategic pattern for dealing with therapeutically indeterminate situations. Using CAM more intensively also resulted in attracting more patients with problems at the edges of medicine.

### Interpretation and comparison with existing literature

In our study, the concept of therapeutically indeterminate situations worked well for interpreting a variety of phenomena usually studied separately under a common rubric. The concept links several lines of research addressing therapeutic behaviors which seem questionable from a strictly scientific perspective. Research in the area of prescription has variously identified “irrational prescribing” [10], “difficult prescribing decisions” [12], or “non-scientific prescribing” [9]. Such interventions are often referred to as “impure placebos” [6, 7] or “non-specific treatments” [5] in surveys on placebo use. While CAM treatments are usually studied separately, they are also typical examples of non-specific treatments in placebo studies [5]. Our study findings support our assumption that these treatments can all be functional equivalents for dealing with a group of problems (which we call therapeutically indeterminate situations). In the accounts of our participants, therapeutic indeterminateness was most frequently associated with minor ailments and medically unexplained symptoms. According to numerous qualitative studies, the incongruence between patients’ symptom presentations and the explanatory models for biomedical disease is a central problem for GPs when dealing with medically unexplained symptoms [20, 21]. While patients usually see their problem as biomedical, GPs see it as originating in psychosocial problems and thus not strictly a medical matter [20, 22]. This leads to frustration for patients as well as GPs, who feel their professional identity is threatened when they are unable to help their patients [20, 23]. Incongruence regarding explanatory disease models plays a minor role in case of minor ailments. But frustration arises also here because patients want a treatment (at least according to physicians), while from a medical point of view, there is little need for action and often no evidence-based effective treatments are available [24].

Our findings suggest that the use of CAM might be the preferred strategic approach of many German GPs when they cannot or do not want to deal with therapeutically indeterminate situations without providing (medical) treatment. The broad use of CAM among German physicians has historical, cultural, legal, and structural reasons [25], and goes hand in hand with wide CAM use among the general population [26]. The pattern of CAM use as well as the broad spectrum of beliefs regarding efficacy in our small sample fit with the findings in national quantitative surveys among GPs [4, 27]. Convinced CAM users no longer or less often feel their professional identity threatened because they have additional specific tools to deal with indeterminate situations. Both convinced GPs and open-pragmatic GPs can use CAM as a relational tool decreasing the incongruence between explanatory models and complying with patients’ expectations. As long as GPs believe – at least to some extent – in the efficacy or plausibility of CAM modalities, these are straightforward and convenient solutions in handling therapeutically indeterminate situations in patients open to such treatment. Among our participants, the deviation from science was not considered a major problem. As in other studies [28, 29], positive personal experiences were the most important justification for using a CAM modality. This was problematic for those of our participants who felt they could not use CAM in an authentic manner on account of their own doubts.

In general, prescribing becomes morally challenging for physicians when they personally think that the treatment is unlikely to do more good than harm. Henriksen and Hansen [11] describe how Danish GPs felt disappointed in themselves when their prescribing conflicted with their ideals. Some GPs compromised their ideals and tried to convince themselves that their prescribing was appropriate. This would also fit with what we refer to as dominance of pragmatism and as stretching of the indication for symptomatic treatment. Possibly, less frequent CAM use might be associated with more frequent use of non-specific and conventional symptomatic treatment. As discussed in the introduction, the use of non-specific treatments among GPs seems more prevalent in the United Kingdom than in Germany [5–7] while CAM use is certainly higher in Germany. Some studies found that GPs using CAM therapies prescribe antibiotics for upper respiratory tract infections less often than those not using CAM [30, 31]. However, such differences can also be explained by other factors (such as differences in patient populations, time, communication training or health system differences).

### Limitations

Our preliminary concept of therapeutically indeterminate situations cannot be considered fully developed. Additional conceptual work and (international) empirical studies are needed to confirm its broader applicability and usefulness. Our decision to restrict ourselves to therapeutically indeterminate situations was arbitrary and followed from our specific study question. A lot of indeterminateness is clearly strongly related to diagnostic uncertainty [15, 32]. Furthermore, we expect that therapeutically indeterminate situations occur in many medical areas, but probably less frequently than in general practice and with somewhat different features. Our study is based on the narrative reflections of the participants. For future projects, it would be desirable to supplement the interviews with direct observations of the actual behavior of GPs. Obviously, our findings are strongly influenced by specific cultural characteristics and the German health system. Germany has a relatively market-oriented health system, a high workload for GPs, very short contact times per patient visit [33], and an unusually wide use of CAM therapies by physicians [4]. We did not fully achieve our goal of identifying factors influencing the preferences for specific strategies. This was mainly due to the dominance of pragmatism and the overall positive attitudes towards CAM, resulting in relatively minor contrasts between subgroups of participants. Yet, the extent of the feeling of responsibility for problems at the edges of medicine along with patient selection among our GP sample emerged as important influencing factors.

## Conclusions

Our preliminary concept of therapeutically indeterminate situations may be useful for understanding why many GPs treat patients in situations where treatment is not unambiguously indicated. It is broader than the concept of medically unexplained symptoms and comes from a different perspective: it does not start with a diagnostic problem but tries to explain therapeutic behavior deviating from normative ideals.

Our study shows that sometimes there is a gap between medical science and patient needs, which the doctors we interviewed are struggling to fill. The different approaches taken by the GPs in our study vary not only from practitioner to practitioner but also for the individual practitioners themselves – either depending on the specific symptoms, the patient’s preferences or the context. Among our participants the more or less convinced use of CAM had an important role when dealing with therapeutically indeterminate situations, while the use of non-specific treatments had a somewhat smaller role.

It was not our intention to provide a prescriptive solution to this issue, rather to illuminate it – to discover what it is that GPs actually do in such situations. There is broad consensus that GPs should manage minor ailments and medically unexplained symptoms more often by making use of empathetic consultation strategies based on a bio-psycho-social approach without providing medical treatments. However, it seems unlikely this is always feasible and sufficient. On these edges of medicine, GPs use highly pragmatic strategies to solve their problems, even if some of these conflict with professional or scientific ideals. Any attempt to resolve this issue must balance these ideals with the realities of general practice and its inherent indeterminateness. We invite GPs to critically reflect how they handle (therapeutically) indeterminate situations in everyday practice.

## Additional file

Additional file 1: Interview guide. (DOCX 14 kb)

## Abbreviations

CAM: Complementary and alternative medicine
GPs: General practitioners
WONCA: World Organization of National Colleges, Academies and Academic Associations of General Practitioners/Family Physicians
