# Supplementary material for: On the edges of medicine – a qualitative study on the function of complementary, alternative, and non-specific therapies in handling therapeutically indeterminate situations
Source: BMC Fam Pract. 2019 Apr 23;20:55. doi: 10.1186/s12875-019-0945-4 (PMC6480714; doi:10.1186/s12875-019-0945-4)
Supplement: Supplementary file 1 — Interview guide. (DOCX 14 kb) [file 12875_2019_945_MOESM1_ESM.docx]

**Complementary, alternative, and non-specific therapies for handling indeterminate situations in general practice – a qualitative study**

**Interview Guide**

**Theme 1: The practice**

Guiding questions: Can you tell us a bit about your practice? Do you have any specialisations regarding patients and therapies? What has changed over the years?

**From here on, address themes in any sequence, or only if they not come up by spontaneously**

**Theme 2: Complementary therapies**

Guiding questions: Do you use complementary therapy in your practice (e.g., homeopathy, herbal medicines, acupuncture etc.)?

Additional questions when the answer is ‘yes’: To what extent? How relevant are these therapies for you? Do you think they are effective? Are they specifically effective or more a placebo therapy? Do think this is scientifically or medically justifiable?

Additional questions when the answer is ‘no’: How do you manage your practice without these therapies frequently used by your colleagues? Why do they need these therapies while you don’t?

**Theme 3: Placebos and non-specific treatments**

Guiding questions: Have you ever used a placebo (e.g. a placebo pill; or a saline injection)? A treatment which you did not consider effective (e.g., a homeopathic remedy if you consider homeopathy a placebo therapy) or not indicated (e.g., an antibiotic for a common cold)?

Additional questions when the answer is ‘yes’: Can you give examples? Why did you do it?

Additional question when the answer is ‘no’: How do you manage practice without?

**Theme 4: Science**

Guiding questions: Which role do science and scientific evidence have for your practical work?

Additional questions, if needed, on the role of experience, plausibility, trials/meta-analyses, should you refrain from a treatment more often?
